# A New Hope for the Treatment of Atrial Fibrillation: Application of Pulsed-Field Ablation Technology

**DOI:** 10.3390/jcdd11060175

**Published:** 2024-06-05

**Authors:** Zhen Wang, Ming Liang, Jingyang Sun, Jie Zhang, Yaling Han

**Affiliations:** 1 College of Medicine and Biological Information Engineering, Northeastern University, Shenyang 110819, China; 2210525@stu.neu.edu.cn; 2 Department of Cardiology, General Hospital of Northern Theater Command, Shenyang 110016, China; lming000919@sina.com (M.L.); sjy2006411039@sina.com (J.S.); cmu2020121913@163.com (J.Z.); 3 National Key Laboratory of Frigid Zone Cardiovascular Diseases, Shenyang 110016, China

**Keywords:** atrial fibrillation, ablation technology, simulation study, pulsed electric field ablation, biological experiment

## Abstract

In recent years, the prevalence of and mortality associated with cardiovascular diseases have been rising in most countries and regions. AF is the most common arrhythmic condition, and there are several treatment options for AF. Pulmonary vein isolation is an effective treatment for AF and is the cornerstone of current ablation techniques, which have one major limitation: even when diagnosed and treated at a facility that specializes in ablation, patients have a greater chance of recurrence. Therefore, there is a need to develop better ablation techniques for the treatment of AF. This article first compares the current cryoablation (CBA) and radiofrequency ablation (RFA) techniques for the treatment of AF and discusses the utility and advantages of the development of pulsed-field ablation (PFA) technology. The current research on PFA is summarized from three perspectives, namely, simulation experiments, animal experiments, and clinical studies. The results of different stages of experiments are summarized, especially during animal studies, where pulmonary vein isolation was carried out effectively without causing injury to the phrenic nerve, esophagus, and pulmonary veins, with higher safety and shorter incision times. This paper focuses on a review of various a priori and clinical studies of this new technique for the treatment of AF.

## 1. Introduction

Atrial fibrillation (AF) is a common cardiovascular epidemic in the 21st century, with its prevalence and incidence increasing with age and varying in different regions. In China, the prevalence of AF is 0.44% in men and women aged 60 years or older, and the prevalence of AF is as high as 7.5% in people older than 80 years. The prevalence and incidence of AF are higher in developed countries than in developing countries [1,2,3,4,5,6]. In the United States, up to USD 26 billion is spent annually on treating AF, with patients with AF spending up to USD 8700 per year on treatment compared to patients without AF. AF affects approximately 6.1 million American adults, and this number is expected to increase, with the number of people with the disease expected to double within 25 years [7,8]. The dangers of AF are that it can lead to thromboembolic complications, which can increase patients’ risk of disability and death. AF significantly increases the risk of ischemic stroke and extracorporeal circulation embolism and is often associated with heart failure, which creates a vicious cycle that increases the risk of death [9,10,11].

The pathogenesis of AF is complex and the quality of life of patients with AF is significantly reduced. European guidelines recommend catheter ablation for patients with medically refractory and heart failure symptoms. It has been shown that pulmonary vein lesions play an important role in arrhythmia and heart rate persistence like AF, making pulmonary vein isolation the cornerstone of catheter ablation [5,12,13,14]. In clinical practice, CBA and RFA are the more commonly used catheter ablation techniques, and clinical studies have shown that RFA produces injury by resistive heating of the tissue and subsequent conduction of heat to deeper tissues. Although the ablation is very effective, it ablates the target area while causing unnecessary damage to other important tissues, such as esophageal damage due to the heat during RFA [15,16,17]. CBA is another widely used ablation modality that is very different from RFA. It ablates the target area by removing heat, resulting in tissue cooling and icing. However, as with RFA, CBA can have several associated complications, and this technique may increase the risk of damage to surrounding tissue structures and increase the incidence of complications such as pulmonary vein stenosis, phrenic nerve injury, and peri-esophageal vagal nerve injury [18,19,20,21,22].

**Figure 1:**
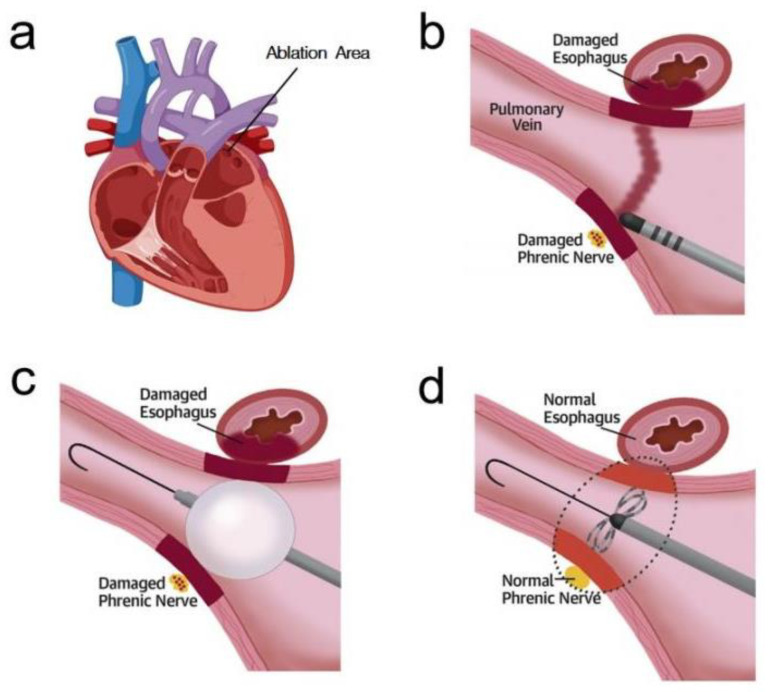
Comparison of damages caused by different ablation methods [27]. (**a**) Coronal diagram of the heart. (**b**) Radiofrequency ablation. (**c**) Cryoballoon ablation. (**d**) Pulsed-field ablation. Some of the models in Figure 1 are available through https://user.medpeer.cn (accessed on 3 April 2024).

Although both ablation modalities are effective in clinical treatment, they produce a range of side effects, and researchers are eager for new techniques to improve the situation. Irreversible electroporation is an emerging technique that has attracted widespread interest and has begun to play an important role in the treatment of cancer, and its technology may also provide new ideas for the treatment of cardiovascular diseases [23,24]. Unlike RFA and CBA, PFA is based on the distribution of an electric field affecting the permeability of the lipid bilayer of its cell membrane, inducing the formation of nanoscale defects or pores in the cell membrane, implying that the cell produces irreversible cell death due to the incompleteness of the cell membrane. PFA will be a new approach to treating AF due to its unparalleled ability to reduce the risk of collateral tissue damage while ensuring the efficacy of ablation [25,26,27,28,29,30,31,32,33,34,35,36,37,38,39]. The comparative results of different ablation modalities are shown in Table 1 and Figure 1. Therefore, this review aims to synthesize and provide up-to-date information on the efficacy and safety of PFA research as it develops, with the ultimate goal of helping to optimize future preclinical studies and advances in ablation technology.

## 2. Pulsed Electric Field Ablation: Simulation Parameter Research

PFA involves the use of electrodes to generate a high-voltage electric field in the target area of the heart by discharging an electric field, which irreversibly perforates the cell membranes of tissues in the target area, increasing cell permeability and thus causing cell death [37]. Differentially complex simulation models may influence the ablation area more. When González-Suárez et al. [38] compared a fully dissected model of the ablation area with a simplified simulation model, the simulation results demonstrated that the electric field was primarily limited to the target site, with very little impact on nearby organs like the lungs and esophagus. At the same time, the difference in the depth of the ablation area between the simplified model and the complete deconvolution model is less than 0.05 mm, which provides data support for future physical field simulations and reduces the complexity of modeling in subsequent studies. The results of the 2D physical field simulation model showed that the ablation zone of the pulsed electric field was confined to the epicardial fat layer, and the width of the ablation zone decreased with the thickening of the fat layer, with the ablation width of the fat layer being about 15 mm when the thickness of the fat layer was 1–2 mm, and then decreased to about 10 mm when the thickness of the fat layer was 5 mm. Meanwhile, it was observed that the neural tissues in the fat had little effect on the ablation zone, but it had an effect on the distribution of the electric field around the area, and as the thickness of the fat layer increased, its electric field distribution reduced. The results suggest that the thickness of fat and the presence of nerves within it may affect the size of the target area for PFA ablation [39].

**Figure 2:**
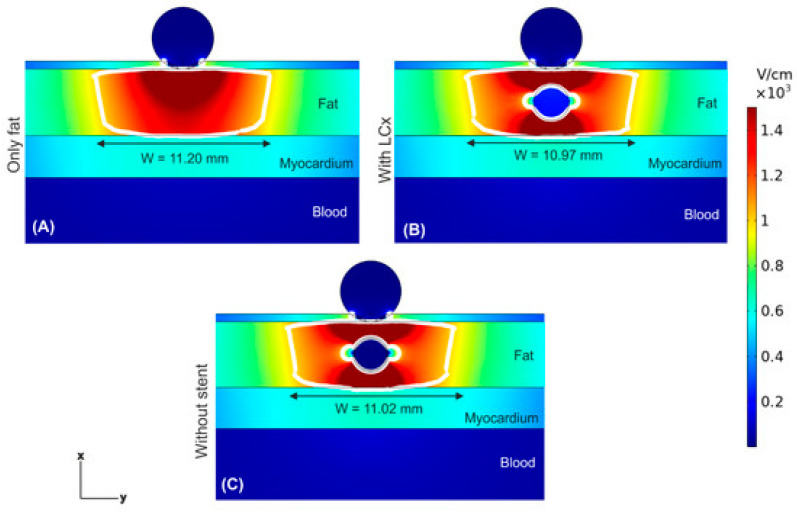
Schematic diagram of epicardial ablation [41]. (**A**) Without coronary artery fat layer. (**B**) With coronary artery fat layer. (**C**) With arterial stent fat layer.

The discharge mode affects the distribution of the electric field in the ablation area. Ji et al. [40] simulated the distribution of the electric field in the myocardium in four discharge modes before and after catheter rotation by using a simulation model including the myocardium, blood, and catheter, and setting up different positive electrodes and negative electrodes, and concluded that widths of the ablated lesions of 10.8 mm, 10.6 mm, 11.8 mm, and 11.5 mm and depths of 5.2 mm, 2.7 mm, 4.7 mm, and 4.0 mm could be formed in the four discharge modes, respectively. The discharge mode will directly affect the electric field distribution in the myocardium, which provides a reference for the subsequent improvement of the design of the pulsed electric field program and the design of the discharge mode of the multi-electrode catheter. During the PFA procedure, the presence of a metallic stent in the coronary artery may affect the normal pulsed electric field distribution, which in turn affects the efficacy of the treatment, and the simulation concludes that the normal pulsed electric field distribution is distorted due to the presence of a metallic stent in the coronary artery. The stent appeared to have a different electric field distribution anteriorly, posteriorly, and laterally; however, the electric field value inside the stent was almost zero. It was also observed that the pulsed electric field ablation region was not affected, and the presence of the metallic stent caused distortion of the electric field but not thermal damage to the adjacent tissue [41], further illustrating the safety of the pulsed-field technique. The simulated ablation effect is shown in Figure 2.

The distance between the applied voltage and the target tissue may affect the depth of myocardial injury. Song et al. [42] used a physical field simulation model to simulate and determine the relevant experimental parameters and tested the typical PFA method directly on the esophagus of New Zealand rabbits, performing pulsed ablation experiments under the parameters: 2000 v/cm and 90 pulses. No luminal narrowing, erosion, or ulceration was found in the esophagus of New Zealand rabbits after 16 weeks of ablation experiments, and PFA has the advantage of not damaging the nearby esophagus during the treatment of AF. The results of Meckes et al. [43] showed that the strength of the electric field of the PFA and the distance to the tissue had a strong correlation with the depth of ablation. The minimum applied voltage required to ablate tissue to produce injury greater than 1 mm was 300 volts. The minimum energy required to achieve a 3-mm depth lesion was 700 volts when contact was made between the catheter and the target tissue, and exponentially higher voltages needed to be applied to achieve the same effect when the distance was increased by 1–2 mm, demonstrating the importance of electrode–target-tissue contact during the ablation process. The safety of the new technique is of paramount importance.

Another study looked at the impact of anisotropic versus isotropic conductivity on PFA characteristics in AF therapy using an anatomically based model of the left atrium and a variety of conductivities and ablation targets. The results showed that the difference in surface ablation area between anisotropic and isotropic conductivity was greater than 73.71%, and the percentage difference in ablation volume size was greater than 6.9%. It was shown that in left atrial ablation, anisotropic conductivity can be used for pulsed-field ablation if the same area and depth are considered [44]. PFA is classified as a non-thermal ablation technique, and the rise in tissue temperature is directly correlated with the heart’s blood flow. Using fluid dynamics and convective heat transfer methods to evaluate the temperature under different pulsed-ablation parameters, the results obtained by fluid dynamics were more accurate, and convective heat transfer predicted lower temperatures than the ablation area in practice, while the temperature of other tissue areas did not exceed the temperature of the ablated area during pulsed-field ablation [37], which once again proved the non-thermal nature of the PFA technique.

## 3. Pulsed-Field Ablation: Biological Experimental Study

The PFA simulation experiments are used to better support the data for clinical studies. Using the relevant data obtained from existing physical field simulation models, ex vivo experiments and animal models are important in setting the background for clinical research.

### 3.1. Animal Experimental Research

Previous AF ablation therapeutic energies have caused cell death through thermal damage. Lavee et al. [45] conducted the first ablation surgery experiments on five pigs using the electric pulse technique, producing permanent non-thermal damage to the target tissues within a few seconds and demonstrating that there was a clear boundary between ablated and non-ablated areas after ablation. Temperature recorders were used to detect temperature changes in the ablated areas during the experiments, and when the hearts of the animals were examined ex vivo 24 h after the procedure, it was found that all lesions had clear boundaries of ablation, and there was no other tissue destruction or charring phenomenon. In this way, it was demonstrated that PFA is a novel, rapid technique without localized thermal effects. Caluori et al. [35] designed a pulsed transmitter and a complete treatment protocol to perform ablation experiments using six sows assisted by different equipment. After a month, the ablation was confirmed, and using finite element analysis software, the various pulse values used in the experiments were simulated and compared with the results. This demonstrated the non-thermal behavior of the ablation technique and simulated the particular physical parameters required in the ablation area. Additionally, the ablation revealed no damage to surrounding tissues, demonstrating the technique’s viability and safety.

**Figure 3:**
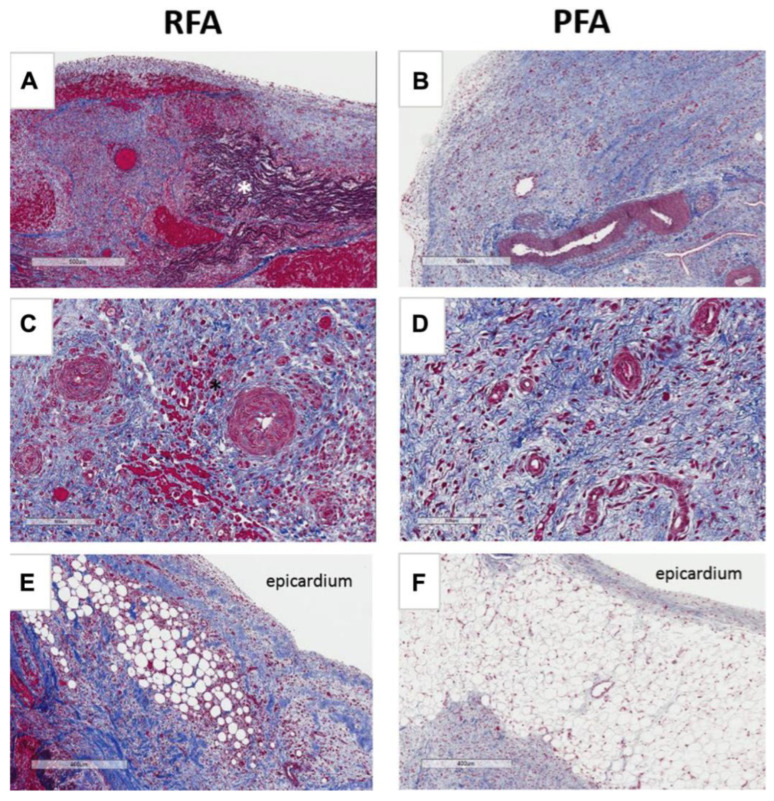
Comparison of different ablation methods [46]. (**A**) Hemorrhage and thrombosis. (**B**) Homogeneous fibrosis and normal patent vessels. (**C**) Scant interstitial hemorrhage. (**D**) Normal arterioles and no hemorrhage. (**E**) Fibrosis and lipogranulomatous inflammation. (**F**) Normal epicardial fat.

To further investigate the safety of the PFA technique, Stewart et al. [46] used a circular pulmonary vein catheter ablation experiment on six pigs using both the RFA technique and the PFA technique. Necropsy verification of the animals using the different techniques after two weeks demonstrated that the use of the PFA technique produced consistent transmural and tissue fibrosis, whereas the use of the RFA technique produced a more severe inflammatory response and some damage to epicardial fat as well as arterioles, proving that the PFA technique can cause fibrotic lesions in tissues without damage to non-target tissues. The ablation effects of RFA and PFA are shown in Figure 3. Koruth et al. [47] observed the electrocardiographic changes in four pigs before and after ablation, and the hearts were dissected and observed after the euthanasia of the animals. No arrhythmia occurred during the ablation process, and the dissected hearts were found to have an average depth and extent of lesions and uniform tissue fibrosis without damage to other tissues.

Considering that the RFA technique is prone to myocardial injury during ablation procedures, Padmanabhan et al. [48] developed a novel catheter system. Seven dogs were used in the experiments, and it was discovered that six of the canines had no more problems following ablation, with lesion regions successfully forming in 20 of the 21 ablation sites. In later follow-up examinations, no harm to the dogs’ pulmonary veins, esophagus, or other structures was discovered. These studies have shown that ablation of ganglionic plexus using direct current PFA is a safe and effective method and that this method causes minimal unnecessary damage. Sugrue et al. [49] conducted experiments on the ablation of Purkinje cells on the surface of the myocardium using the PFA technique and monitored the changes in the electrical signals of the left ventricular cardiomyocytes and the Purkinje cells in the experiments, which showed that the degree of damage to the Purkinje cells was related to the duration and the pulse voltage of the electrical pulse technique. Repeated measurements of ECG signals at 30 days did not reveal significant changes in myocardial amplitude, and pathologic tissue confirmed the targeted nature of the pulse ablation technique.

PFA is an ablation technique with potential safety advantages over RFA. Koruth et al. [50] examined their electrophysiological and histological effects in a preclinical study and compared the safety and feasibility of isolating the pulmonary vein and the superior vena cava between the different waveforms of RFA and PFA. The results showed that all target veins using the PFA technique were isolated for the first time, the effect of pulmonary vein isolation was higher than that of the control RF group, and the stenotic condition of the pulmonary veins occurring in the RF group was not detected in the pulsed ablation group, which provides a scientific basis for the validity and safety of the separation of the superior vena cava from the pulmonary veins in the clinical setting. Yavin et al. [51] designed a novel PFA system with the ability to adjust the shape of the catheter tip electrode according to the clinical situation. Experiments were conducted using 16 pigs to compare the damage to the esophagus and phrenic nerve by the RFA technique, and one month after ablation the ablation damage persisted in 91.7% of the pigs, and 97.8% of the ablated areas showed transmural lesions. No effects on the esophagus and phrenic nerve were found with the PFA technique, demonstrating the safety and efficacy of this technique.

As a novel non-thermal ablation modality, the wall penetration ability of PFA ablation has an important impact on the therapeutic outcome of AF. Varghese et al. [52] performed PFA ablation in 10 pigs by histologically examining the experimental pig hearts in sections and using stains to detect the wall penetration of each lesion. The results showed that both left and right atrial ablation lesions were permeable to the wall, with a maximum thickness of 8–12 mm of ablated tissue, and sectioned histology revealed a highly uniform width of the lesion area and no arrhythmias or other complications during the procedure. A similar study was conducted by Doshi et al. [53] where PFA ablation was performed on 10 pigs and the experimental pigs were divided into two subgroups: 8 experimental pigs were debrided 2 h after ablation and 2 experimental pigs were debrided 30 days after ablation. The permeability of each lesion was examined using staining. The results showed that there was no significant difference in the depth and width of the ablation zone between the different groups (depth: 5.65 mm and 5.68 mm, width: 15.68 mm and 16.98 mm). This study demonstrates that pulsed electric field ablation produces consistent permeable lesions.

For a substantial summary as well as a comprehensive comparison, other relevant animal studies of the PFA technique are summarized in Table 2. All studies, despite different catheter electrode configurations as well as different ablation modalities, reported on the efficacy and safety of this new technique.

### 3.2. Clinical Trial Research

As a novel technique for the treatment of AF disease, it has been documented that PFA technology produces irreversible electroporation in cell membranes. Previous ablations have relied on the ablation of a heat source conducted over time, whereas this ablation is an undifferentiated ablation of all tissues. The PFA technique has several potential advantages, allowing for the selective ablation of myocardial tissues during the ablation process and a very short ablation time, avoiding the risk of pulmonary vein stenosis.

**Figure 4:**
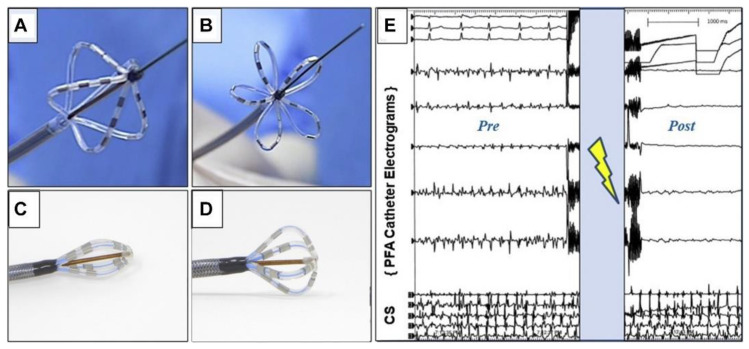
PFA ablation device [63]. (**A**–**D**) Schematic diagrams of the ablation catheters in different attitudes. (**E**) Ambulatory electrocardiogram before PFA ablation and ambulatory electrocardiogram after PFA ablation.

Reddy et al. [27,62,63] treated 22 patients with AF using the PFA technique; pulmonary vein isolation was achieved in all patients and the average procedure time was one hour. The potential benefits of PFA were verified by the absence of complications in all patients. A total of 81 patients were treated with ablation using a proprietary pulse waveform, and the effect of pulmonary vein isolation was examined using electrophysiologic techniques. It was found that all patients achieved pulmonary vein isolation after 3 months, and no adverse events such as phrenic nerve injury or pulmonary vein isolation were detected during the subsequent 120-day follow-up, validating the tissue selectivity of the PFA technique during ablation. In a later study of patients with persistent AF, no protective measures were taken for the surrounding tissues during the treatment, and electrophysiological follow-up of the ablated patients was performed 3 months later to verify the clinical results, which showed that pulmonary vein isolation was achieved in all 25 patients, and there was no pulmonary vein stenosis and no esophageal or phrenic nerve injury. The uniqueness of the PFA technique determines the success rate of pulmonary vein isolation and the safety and durability of the periphery of the ablated tissues. This study also extends the PFA technique from paroxysmal AF to persistent AF, laying the foundation for the clinical management of cardiac arrhythmias. The ablation device and pre- and post-ablation ECGs are shown in Figure 4.

To verify whether the PFA technique would affect the myocardium differently compared to thermal ablation, Reddy et al. [64] adopted a new compressible 9-mm tip ablation catheter that can switch between two types of energies, radiofrequency and electric field, which is the first time that the catheter has been used in a clinical setting. A total of 76 patients with AF were treated in this clinical study, of which 40 patients with AF were ablated with radiofrequency/pulsed electric field and the remaining 36 patients with AF with pulsed electric field/radiofrequency, with an average ablation time of 22.6 min. No phrenic nerve injury or esophageal injury occurred after ablation. It is concluded that this novel catheter can be ablated safely and rapidly using multiple ablation modalities or a fully PFA approach. Ablation will be more effective and safer with a different approach compared to areas requiring greater depth of wall penetration and certain areas closer to other tissues. The most desirable approach may be to utilize a combination of the safety of PFA and the clinical experience of RFA in clinical treatment. Yosuke et al. [65] conducted a comparative study of different ablation modalities, where 41 patients were medically examined before and after ablation. A total of 18 of the patients were treated with PFA and 23 patients were treated with a thermal ablation procedure. The postoperative changes in myocardial tissue were more homogeneous in patients with PFA, and there was no postoperative vascular injury or intimal hemorrhage. No delayed myocardial enhancement was found in patients treated with PFA in the postoperative period, concluding that the PFA technique produces a more complete injury to the target myocardium compared to the thermal ablation technique.

The durability of the PFA technique for the treatment of AF has received attention from clinical researchers. Verma et al. [66] first applied a circular multi-electrode array catheter in a clinical study. Thirty-eight patients with paroxysmal or persistent AF were recruited in this study. The results of the technique’s pulmonary vein isolation were evaluated 30 days after the procedure, and pulmonary vein isolation was accomplished in all of the patients. No serious adverse events related to the PFA technique, such as phrenic nerve injury, esophageal injury, or death, were observed during the 30-day follow-up. Gunawardene et al. [67] performed pulmonary vein isolation experiments in patients with AF using multiple types of ablation catheters, and the clinical results showed that pulmonary vein isolation was successfully performed in all 20 patients and that there was no complex electrocardiographic segmentation of the edges of the lesion area after ablation. The finding that the ultrahigh-density mapping PFA technique can perform pulmonary vein isolation in the posterior wall of the left atrium, and at the same time, the probability of pulmonary vein reconnection occurring at an early stage is extremely low shows that this is a promising new technique for the treatment of AF. Loh et al. [29] explored the feasibility and safety of the single-pulse PFA technique for pulmonary vein isolation in patients with AF, a single-pulse ablation pulmonary vein isolation study was performed in 10 patients with paroxysmal or persistent AF, and the results showed that all 40 pulmonary veins were successfully isolated without reconnection; this is the first study in which pulmonary vein isolation was accomplished by single-pulse ablation.

Catheter ablation procedures for AF may cause complications and damage to other tissues, and clinical data suggest the tissue-selective specificity of the PFA technique. Cochet et al. [68] recruited 41 patients with paroxysmal AF for pulmonary vein isolation using three treatment methods, namely, RFA, CBA, and PFA, and assessed esophageal and aortic injuries using myocardial delayed enhancement. The ablation site was in contact with the esophagus in all patients, and postprocedural results showed no esophageal lesions—which are common with thermal ablation methods—in patients undergoing PFA treatment, confirming the unique tissue specificity of the PFA technique. Some patients developed transient aortic injury after both PFA and thermal ablation treatments, but the pathologic significance was unclear. These findings illustrate the tissue-selective nature of PFA.

Numerous clinical studies have demonstrated the favorable safety profile of PFA relative to other ablation techniques, but this technique is associated with adverse symptoms produced by other ablation modalities during the treatment of AF. Gunawardene et al. [69] reported the first case of arterial spasm during pulsed electric field ablation. They alleviated the phenomenon with nitroglycerin during the procedure, and the patient did not experience postoperative adverse symptoms such as angina pectoris. During epicardial ablation, Reddy et al. [70] found that when ablation therapy is performed away from the coronary arteries, it does not cause coronary artery spasms during the ablation, while when the ablation energy is delivered to the vicinity of the coronary arteries, it usually causes arterial spasm, which can be relieved by taking nitroglycerin. Renal injury due to ablation therapy is another uncommon negative effect. Mohanty et al. [71] divided 103 patients into two groups based on different post-procedure methods after PFA ablation for AF and observed that the number of PFA applications and the post-procedure hydration therapy seem to be important predictors of lung injury; therefore, renal injury can be prevented by injecting enough fluids immediately after ablation. Another study by Venier et al. [72], which investigated 68 patients with AF who were treated with PFA, obtained the same results. Additionally, the number of PFA applications seemed to be an important factor in the severity of hemolysis.

## 4. Discussion and Analysis

As a novel approach to AF ablation therapy, the development and application of PFA technology have aroused great interest. PFA technology is a non-thermal ablation technique. In previous catheter ablation techniques for AF, radiofrequency or cryogenic energies were usually used, and such energies may cause serious complications. The principle of PFA technology is to apply high-voltage electrical pulses to the phospholipid bilayer of the cell membrane, leading to the formation of transmembrane potential, causing changes in cell membrane permeability that in turn disrupts the homeostasis of the intracellular environment and leads to cell death. The electric field is usually formed between two or more electrodes of the catheter, and the greater the applied field strength the greater the effect on the target tissue. Due to the low field strength threshold required for irreversible electroporation to occur in cardiomyocytes, cardiomyocytes have a high sensitivity to the electric field, and due to its ability to produce transmural, sequential damage by generating irreversible electroporation, PFA has advantages such as safety and efficacy, which other ablation techniques do not have.

### 4.1. Discussion

AF is the most common arrhythmia symptom, and pharmacologic treatment of AF has been the mainstay of therapy for decades; however, limited therapeutic efficacy coupled with incomplete evaluation of other symptoms caused by AF has led to a tremendous development of ablative therapies [2,3,4,5,6,7,8,9,10]. Studies have demonstrated that pulmonary vein lesions play an important role in cardiac heart rate maintenance, thus pulmonary vein isolation has become a new technique for the treatment of AF, and catheter ablation has been shown to have a significant effect on pulmonary vein isolation [5,10,12,13,14]. RFA and CBA are the more common therapeutic means, and clinical data have shown that there is no significant difference between the different therapeutic techniques in the treatment of AF. However, since both ablation techniques are heat source ablation techniques, they are prone to causing other tissue damage during treatment, for example, pulmonary vein stenosis and phrenic nerve injury [15,16,17,18,19,20,21,22].

Researchers are looking for a new technology to be used in clinical studies of AF treatment. PFA technology has attracted extensive research interest and has shown relatively good results in cancer treatment. RFA technology converts low-voltage, high-frequency electrical energy into thermal energy to perform fractional ablation at the target lesion; CBA technology releases liquid refrigerant N20 from a catheter-tipped balloon into the tissue of the lesion to perform ablation. For the PFA technology, the electric field energy delivered by the catheter is used to damage the phospholipid bilayer of the cell membranes of cardiomyocytes, changing the permeability of the cell membranes and causing the destruction of the intracellular homeostatic environment, hence treating AF. Since the tissues adjacent to cardiomyocytes have different thresholds of pulsed-field strength, PFA technology has the advantage of tissue selectivity that other ablation techniques do not have; this avoids potential damage to other tissues around the ablation object [23,24,25,26,27,28,29,30,31,32,33,34,35,36,73].

Physical field parameter simulation research is an important stage in the clinical application of new technologies, and reasonable PFA parameters will substantially reduce the cost of clinical research [37,44,74]. The researchers obtained a simplified cardiac model from between models of different complexities that can get more accurate simulation results, which provides data support for reducing the complexity of the model in the future. The effects of cardiac fat thickness and arterial metal catheters on the distribution of electric fields were investigated in subsequent studies, and these simulation results had a positive effect on the treatment of the disease in AF patients with different conditions [38,39,41]. The different discharge patterns of the catheters directly affect the distribution of the electric field in the myocardium, and an increase of 1–2 mm in the contact distance between the catheter and the tissue requires a doubling of the field strength to achieve the same ablation effect [40,43]. In another study, it was found that the use of hemodynamics resulted in a normal ablation process without thermal damage to other tissues. After the physical field simulation study, some researchers used animal experiments to verify the reliability of the simulation data and found that PFA does not cause negative damage to the esophagus and other tissues, illustrating the safety and reliability of this technology [37,42].

Simulations from different perspectives are providing a solid database for subsequent clinical studies, and a large number of simulations will be needed in the future for more detailed studies of different patient symptoms. Simulation data on simplified cardiac models reduces the modeling complexity for subsequent studies and improves simulation efficiency. However, no researchers have yet conducted a detailed study of coronary stents in patients with AF to see whether the mesh morphology of the stents distorts the electric field distribution during PFA therapy. This may require researchers to conduct further studies to fill the data gaps in simulation models of different complexities.

The results of animal experiments play a huge role in the promotion of clinical applications. A large number of researchers have conducted animal experiments and found that PFA has good therapeutic effects. For example, researchers recorded temperature changes during ablation experiments and found that the boundaries of the ablated area were clear without tissue scorching or damage after the postoperative ex vivo debridement. In another study, it was found that the fibrosis of the lesion tissue was uniform after ablation, and the ablation depth and width of the lesion area were uniform and consistent, with no damage to non-targeted tissues [35,45,46,47,75]. Since then, several animal experimental studies on different animals have demonstrated that the PFA technique has good tissue specificity and can effectively perform pulmonary vein isolation for AF treatment. It avoids damage to esophageal stenosis and phrenic nerve injury caused by the use of RFA or CBA in the treatment of AF. Some researchers have also found that the PFA technology has better transmural damage ability than thermogenic ablation and can effectively block the reconnection of pulmonary veins compared with other ablation methods. At the same time, researchers have found that the PFA technique has better transmural damage ability than thermal ablation, and the lesion area has a high degree of consistency in regional width, homogeneous tissue fibrosis in the region, and is able to block the reconnection of the pulmonary veins more effectively than other ablation methods [35,43,48,49,50,51,52,53,54,55,56,57,58,59,60,61].

As a new treatment for AF, animal testing is a necessary preclinical stage. The ultimate application of this technology will be in the treatment of patients with AF, and we can find information that this technology is being tried in the clinic. Because of the special characteristics of the human body, it may be more useful to conduct experiments on larger mammals, such as pigs and large dogs, to guide clinical results. However, this can be a costly burden for researchers, so the new technique of ex vivo animal tissue experiments may have a positive significance for animal experiments and preclinical studies.

The fundamental purpose of physical field simulation studies and animal experiments is to provide theoretical support for the clinical study of the PFA technology. Unlike previous heat source ablation, PFA technology causes cell death by creating nanoscale voids in the cell membrane. Researchers conducted a clinical study of PFA in 22 patients, all of whom had successful isolation of the focal pulmonary vein, with an average procedure time of one hour and no complications. In a follow-up study, the researchers used a proprietary pulse waveform to successfully isolate all of the targeted pulmonary veins. At follow-up, there was no phrenic nerve injury or esophageal injury, except for one case of pericardial tamponade, and the 12-month arrhythmia-free Kaplan–Meier estimate averaged 87.4% [27,62]. The safety and sustainability of the PFA technique for the treatment of persistent AF and ablation of the posterior wall of the left atrium have been of interest to researchers. Cardiac computed tomography of the esophagus in 25 patients after the procedure showed no mucosal lesions or pulmonary vein stenosis; this study extends the application of the PFA technique from paroxysmal AF to the treatment of persistent AF [63].

To better compare the advantages and disadvantages of thermogenic ablation and PFA, several researchers have conducted in-depth comparisons of the two techniques using clinical trials. Patients treated with PFA were found to have more homogeneous lesions in the myocardial tissue, and there was no damage to tissues such as blood vessels. Similar results were obtained in another study, and the researchers suggested that combining the safety of PFA with the clinical experience of RFA may be a safer and more effective way to treat AF, as PFA can control the ablation area of the target lesion and has a very low likelihood of reconnecting the pulmonary veins at the end of treatment. Similar findings were reported in another study comparing the three treatment modalities of RFA, CBA, and PFA, in which the PFA technique did not cause symptoms such as esophageal injury and avoided aortic injury relative to the thermogenic ablation technique, findings that illustrate the tissue-selective specificity of PFA [64,65,66,67,68]. In a follow-up study, hundreds of patients with paroxysmal AF were randomized to different ablation modalities to compare the safety of the three treatment modalities, RFA, CBA, and PFA, and after one year, a similar proportion of patients with PFA as well as patients with thermal ablation were found to have achieved the primary efficacy endpoint. Several studies have demonstrated the favorable safety profile of PFA [76,77,78]. A similar study was conducted to evaluate the safety and durability of pulsed-field ablation of AF in a subgroup of 72 patients. The safety and durability of the treatment of AF were comparable to the results of thermal ablation at 6 months, but with a shorter learning curve; the results of this study have been confirmed by other researchers [79,80].

There have been cases where symptoms of arterial spasm may occur during PFA procedures for AF, but numerous studies have found that this symptom does not only occur during PFA but also during RFA and CBA for AF, where it is a rare symptom. In a study of 2913 patients, the arterial spasm was found to occur in only nine cases (approximately 0.31%), and although the chances of this symptom occurring are small, careful monitoring of the patient’s electrocardiogram during the procedure is necessary to allow for rapid resolution of this symptom [69,70,81,82]. It has been found that there is a small chance of kidney injury during ablation for AF, and there appears to be a relationship between this kidney injury and the type of ablation. In one study, RFA and CBA were found to cause a greater chance of kidney injury than pulsed-field ablation, 4.3% versus 1.8%, respectively, whereas in the subgroup that used pulsed-field therapy for AF, there was only a 1% probability of kidney injury, which may also be due to the small number of patients in the pulsed-field subgroup (306). It was also found that it was possible to prevent symptoms of renal injury by using hydration therapy in the immediate postoperative period occurrence [71,72,83,84]. These studies demonstrate that PFA may cause less unwanted harm, such as coronary artery spasm versus kidney injury, relative to other ablation methods. In the future, it will be interesting to see whether the development of PFA technology can completely avoid these unnecessary injuries.

Epicardial ablation has attracted a lot of attention from researchers, mainly because the beating of the heart is governed by the autonomic nervous system. The ganglionic plexus of the epicardium is an important part of this nervous system, and these plexuses are usually located in the fatty layer of the epicardium. Researchers used a computerized physical simulation to study the effect of the ganglion plexus on the ablation area and observed that the plexus had an effect on the width of the ablation. The researchers modified the ablation device, obtained the ablation parameters in a computer simulation model, and performed epicardial ablation in experimental pigs. The results showed that the modified ablation device was effective in ablating epicardial ganglion from the ablation treatment and that it minimized the damage to the myocardium and collateral structures. In one clinical study, ganglion ablation was found to be successful in all patients, with no complications at one-year follow-up. In another study comparing RFA versus PFA, it was found that PFA is an exciting new technology; it was also found that PFA may be more friendly to certain nerves, allowing for this selectivity to be fully utilized, providing unique opportunities for ganglion-subtraction ablation. While the results of ganglion subtraction from ablation appear to be favorable in the short term, the long-term safety and efficacy of ablation are still worth exploring [41,85,86,87,88,89].

Finally, although the PFA technique is safe and effective in animal experiments, which may depend on the findings of various parameters of the physical field simulation, the optimal treatment protocols and device parameters still need to be further studied and refined. In particular, some device parameter variables in the new technique, such as pulsed-field strength, number of pulses, pulse duration and frequency, etc., need to be considered [25,72,90]. A review of the safety of PFA ablation is shown in Table 3. In addition, the development of PFA technology also requires further research and parameterization of pulsed devices and catheters, including catheter diameter and shape, amount of contact with ablated tissue, and number of electrodes. In future clinical studies, good pulsed ablation devices and reasonable ablation parameters may be more favorable for the application of this technology in AF treatment. In the future, a large number of clinical trials will demonstrate the safety and efficacy of PFA technology compared with other thermal ablation techniques in the treatment of AF. With the improvement of treatment protocols and the acquisition of a large amount of clinical data, PFA technology may be accelerated into clinical application [77,78,79,91].

### 4.2. Development Direction

Studies have demonstrated the effectiveness and safety of the PFA technique in the treatment of AF, and some researchers have compared thermal ablation and PFA in clinical settings, with clinical results showing that non-thermal ablation is safer and more effective in treatment. However, due to the insufficient number of such comparative trials, researchers need to conduct further studies on this technique to prove its advantages.

The PFA parameters in this technique have an important impact on AF treatment, and the ablation parameters in clinical application need to be further investigated, for example, the number of pulses and the ratio of pulses and pulse duration, which have a direct impact on the depth and width of the ablation area. In addition, as a new technology, more clinical studies and follow-up studies are needed to prove the safety and efficacy of this technology.

Finally, is it possible to apply the clinical experience of thermogenic ablation to the PFA technique, and it may be possible to combine the two techniques in clinical applications for better therapeutic results. There are studies demonstrating that the PFA technique can be effective in the treatment of paroxysmal and persistent AF therapy, and it might be an interesting study to combine clinical data with this technique for the permanent treatment of AF.

## 5. Conclusions

Based on a large number of physical field simulation studies and animal experimental results, PFA has demonstrated greater safety and efficacy in AF treatment compared to RFA and CBA. These studies found that PFA was effective in producing well-defined ablative injuries and minimizing the risk of collateral damage. Additionally, without taking the protection of other adjacent tissues into account, PFA has good ablation specificity and does not cause damage to the esophagus or phrenic nerve. In addition, the results of a clinical study showed that the probability of reconnection of the pulmonary veins after treatment with PFA technology was extremely low and complications were not detected during patient follow-up. This suggests that the PFA technique will be a promising tool for the treatment of AF. Finally, it is worth mentioning that the PFA technique was developed for treating other diseases, and the possibility of using this technique for a wider range of disease treatments in the future deserves to be explored through further research.

## Figures and Tables

**Table 1:** Comparison of different ablation methods.

Reference	Ablation Mode	Energy Source	Ablation Process	Characteristic
[30,31]	Radiofrequency ablation	Heat energy generated using low-voltage, high-frequency electrical energy	The catheter is delivered to the site of the lesion, releasing energy to cause partial myocardial degeneration and necrosis.	Low efficiency of spot ablation and long learning curve for the operator
[32,33]	Cryoballoon ablation	Liquefied refrigerant in the balloon liner	Evaporation of liquefied refrigerant by heat absorption, resulting in local tissue necrosis due to reduced temperature at the ablation site	The ablation effect is still dependent on balloon apposition and lacks tissue selectivity
[34,35,36]	Pulsed-field ablation	High-voltage, high-frequency multi-electrode pulses generate non-thermal energy	The electric field is applied to the phospholipid bilayer of the cell membrane for a short period of time, and irreversible penetrating damage is formed in the cell membrane.	Non-thermal energy, strong tissue selectivity, no harm to nearby tissues

**Table 2:** Animal studies on other related pulsed electric field ablation techniques.

Reference	In Vitro/In Vivo	Subject	Energy	Catheter Style	Summary
[54]	In Vivo	Swine	Not mentioned	Circular type	There was no sign of thermal damage after ablation, the lesion was continuous in extent, and the thickness of the lesion increased with increasing energy.
[55]	In Vivo	Swine	Not mentioned	T-type	There is a clear relationship between the energy produced by the electrodes and the area of the myocardial tissue lesion.
[49]	In Vivo	Swine	500 V	Circular type	Ablation produces fibrotic lesions with acute electrical effects and does not damage non-targeted tissue.
[56]	In Vivo	Swine	400–800 V/cm	Balloon type	Selectively affects cardiac myocytes but not vascular and neural tissue.
[57]	In Vivo	Swine	1600 V/cm	Petal type	It effectively blocks electrical activity from the pulmonary veins to the atria with myocardial contraction and does not cause pulmonary vein stenosis.
[58]	In Vivo	Rabbit	50–500 V	Circular type	The endocardium is most susceptible to electroporation and may contribute to arrhythmia susceptibility.
[43]	In Vivo	Rabbit	2000 V/cm	Self-developed	No significant stricture, erosion, or ulceration of esophageal tissue was observed.
[59]	In Vitro	Rat	400 V/cm690 V/cm	Not mentioned	Cell type is selectively specific for electroporation production but electrode proximity to the target tissue is still important for efficacy.
[60]	In Vitro	Rat	1000 V/cm1200 V/cm	Not mentioned	Compare different cell types within the cardiovascular system and determine the optimal voltage threshold for selective ablation of cells.
[61]	In Vivo	Canine	Not mentioned	Straight type	Transepicardial treatment of atrial fibrillation with DC current is feasible as an adjunct to pulmonary vein isolation.

**Table 3:** Summary of additional safety assessments (N = 121) [90].

	No. of Patients Assessed	Findings
Esophagus Findings		
EGD	38	No esophageal lesions
CMR	18	No esophageal enhancement
Phrenic nerve		
Fluoroscopy at end of procedure	121	No paresis/palsy
Fluoroscopy at 3 months	110	
Brain MRI	18	16 of 18 (89) DW-negative
PV stenosis		
EAM at 3 months	110	No PV stenosis or narrowing
CT at 3 months	74	

EGD: esophagogastroduodenoscopy; CMR: cardiac magnetic resonance; MRI: magnetic resonance imaging; PV: pulmonary vein; EAM: electroanatomical mapping; CT: computed tomography; DW: diffusion-weighted.
